# Untangling the positive genetic correlation between rainbow trout growth and survival

**DOI:** 10.1111/j.1752-4571.2012.00251.x

**Published:** 2012-11

**Authors:** Harri Vehviläinen, Antti Kause, Hanna Kuukka-Anttila, Heikki Koskinen, Tuija Paananen

**Affiliations:** 1 Genetics Research, MTT Agrifood Research FinlandJokioinen, Finland; 2 Department of Biosciences, University of HelsinkiHelsinki, Finland; 3 Finnish Game and Fisheries Research InstituteHelsinki, Finland; 4 Tervo Fisheries Research and Aquaculture, Finnish Game and Fisheries InstituteTervo, Finland

**Keywords:** animal breeding, aquaculture, body size, evolutionary theory, fitness cost, life-history trade-off, *Oncorhynchus mykiss*, quantitative genetics

## Abstract

Explanations for positive and negative genetic correlations between growth and fitness traits are essential for life-history theory and selective breeding. Here, we test whether growth and survival display genetic trade-off. Furthermore, we assess the potential of third-party traits to explain observed genetic associations. First, we estimated genetic correlations of growth and survival of rainbow trout. We then explored whether these associations are explained by genetic correlations with health, body composition and maturity traits. Analysis included 14 traits across life stages and environments. Data were recorded from 249 166 individuals belonging to 10 year classes of a pedigreed population. The results revealed that rapid growth during grow-out was genetically associated with enhanced survival (mean *r*_G_ = 0.17). This resulted because genotypes with less nematode caused cataract grew faster and were more likely to survive. Fingerling survival was not genetically related to weight or to grow-out survival. Instead, rapid fingerling growth made fish prone to deformations (*r*_G_ = 0.18). Evolutionary genetics provides a theoretical framework to study variation in genetic correlations. This study demonstrates that genetic correlation patterns of growth and survival can be explained by a set of key explanatory traits recorded at different life stages and that these traits can be simultaneously improved by selective breeding.

## Introduction

Genetic constraints for multitrait evolution are typically expressed as negative genetic correlations between traits: a genetic increase in one trait is associated with a decrease in the other. The existence of negative genetic correlations between fitness components is essential for the development of life-history theories and optimization models, and they set severe limitations on plant and animal breeding programmes (Lande 1979; Schluter 1996; Phillips et al. 2001). This is because genetic constraints restrict phenotypic space available for evolution (Gasser et al. 2000; Pigliucci and Kaplan 2000; Brakefield 2006).

Negative genetic correlations, that is, genetic trade-offs, between fitness component traits have been suggested to evolve via erosion and fixation of alleles owing to directional selection (Roff 1996; Merilä and Sheldon 1999) and resource allocation trade-offs (van Noordwijk and de Jong 1986). Although the existence of such negative genetic correlations has been unambiguously demonstrated in variance component studies and by genetic trends in animal breeding programmes, positive genetic correlations are still found. In fact, in the review by Roff (1996), an unexpected 60% of the genetic correlations between fitness component traits are positive. Similarly, Carlson and Seamons (2008) found in their review that the distribution of published genetic correlations between fitness-related traits of salmonids is heavily skewed towards positive values (median *r*_G_ = 0.32). Accordingly, the negative genetic correlations are not prevalent, and alternative explanations must exist.

Rapid growth and thus higher body weight at a time of maturation are important components in organisms’ life history (Roff 1992). Rapid growth often exhibits genetic costs owing to limited resources allocated across competing body functions (van Noordwijk and de Jong 1986). The mechanisms behind the costs of rapid growth are not fully understood (Dmitriew 2011). Nevertheless, plausible physiological costs can arise owing to metabolic requirements and energy-use efficiency of rapidly growing individuals (Blanckenhorn 2000; Teuschl et al. 2007; Dmitriew 2011). Thus, even though growth is not a direct fitness component itself, negative genetic correlations between growth and fitness component traits such as survival can be expected. On the other hand, ‘overall vigour’ is sometimes observed as positive genetic correlations between growth and fitness components. For instance, in farm animals, growth and survival are sometimes positively correlated (e.g. Chicken: de Greef et al. 2001; Lambs: Riggio et al. 2008; Pigs: Roehe et al. 2010). Accordingly, there is a need for increased understanding why genetic correlations between growth and fitness components are sometimes positive and sometimes negative and what causes the variation in the sign.

Similar to evolution in wild, genetic trade-offs constrain selective breeding programmes. Evolutionary genetics provides a theoretical and experimental framework to understand and study the causes of variation in genetic correlations. We used this framework to analyse data from an aquaculture breeding population to assess the degree of genetic trade-off between growth and survival and whether the observed correlation can be explained by other traits, such as those related to animal health. This information can be applied to select for balanced animals with improved production without compromising survival and animal heath. For the evolutionary null hypothesis, we assumed that rapid growth imposes a genetic cost on survival. Ecological costs (e.g. predation and limited nutrition) can possibly mask the cost of growth on survival (Dmitriew 2011). In our setting, these costs are minimized, but fish still face a multitude of mortality factors. We analysed (i) the genetic association of growth and survival in different life stages and environments using data from 249 166 individual rainbow trout, Salmonidae: *Oncorhynchus mykiss* (Walbaum), originating from 10 year classes of a pedigreed population. Furthermore, we explored (ii) possible explanations for the associations of growth and survival by estimating genetic correlations between survival and a set of health, maturity and body composition traits.

The results of our study have direct application to aquaculture breeding programmes. Rapid growth rate is typically the first trait to be selected when a new breeding programme is established, and subsequently other economically important traits such as maturity age and product quality are added to a selection index. However, prolonged selection solely for growth and production traits may lead to serious problems such as increased disease susceptibility and malformations (Rauw et al. 1998). Because of its impact on production efficiency, industry profit and reduced environmental load (Kause et al. 2006; Quinton et al. 2007), high selection emphasis on growth rate will remain in the foreseeable future. Therefore, controlling the correlated effects of growth selection on other traits is crucial for long-term sustainability of breeding programmes, and there is increased interest to select for survival, health traits and resistance or tolerance to specific diseases. Moreover, our study examined indicator traits that can be used to select for increased survival. Survival is a binary trait, and it is not possible to distinguish genetically superior individuals from the general surviving population based on phenotype alone. Indicator traits that genetically correlate with survival, for example parasite/parasitoid counts or skeletal malformations, increase the accuracy of identifying individuals’ genetic potential for survival (Hazel 1943). In our study, we utilize evolutionary theory to unravel genetic architecture of growth and survival. The results can be used in breeding programme to find suitable combinations of traits to be selected in a balanced breeding goal, that is, production and survival traits and underlying health traits causing variation in the genetic correlation between these two. This will lead to better animal welfare and to more environmentally sustainable aquaculture production.

## Materials and methods

### Experimental design

Ten year classes of rainbow trout were monitored for 14 growth, survival, health, body composition and maturity traits through their 3-year life cycle from juvenile fingerlings to adult fish. In each year class, 109–341 full-sib families were produced via nested paternal or partial factorial mating protocols. To assess the genetic relationship between growth and survival, survival until harvest and body weights at three ages were recorded. Additionally, underlying traits of cataract caused by parasitic nematode, skeletal deformations, flesh colour, entrail percentage and maturity age were recorded after two growing seasons. These data originated from the Finnish national rainbow trout breeding programme maintained together by the Finnish Game and Fisheries Research Institute (FGFRI) and MTT Agrifood Research Finland. The freshwater breeding nucleus is held at FGFRI Tervo Fisheries Research and Aquaculture station in Central Finland.

### Population structure

Survival records were obtained during two life-cycle stages: a juvenile fingerling period (survival from initial body weight of 2 g until body weight of 50 g) and a grow-out period (from 50 to 1000 g). The analysis consisted of observations of 249 166 individuals. The fish originated from three subpopulations consisting of 10 year classes belonging to four generations (Table 1). Each year class consisted of 109–341 families of 48–168 sires and 79–272 dams, mated using either nested paternal or partial factorial designs. A total of 1159 ancestors without observations and born in 1989, 1990, 1992 and 1993 were included to complete the pedigree.

**Table 1:** Population structure and mating designs in each year class

Population/generation	Fertilization year	No. of sires	No. of dams	Mean (range) dams per sire	Mean (range) sires per dam	No. of full-sib families	No. of family tanks	No. of sea test stations
Population I								
1	1995	92	272	3.0 (1–5)	1.0 (1–1)	272	370	–
2	1998	71	128	1.8 (1–4)	1.0 (1–1)	128	132	1
3	2001	121	154	2.5 (1–6)	2.0 (1–3)	303	303	2
4	2004	130	93	1.9 (1–5)	2.7 (1–4)	250	250	2
Population IIa								
1	1996	75	150	2.0 (1–4)	1.0 (1–1)	150	150	1
2	1999	48	109	2.3 (1–4)	1.0 (1–1)	109	150	2
3	2002	113	139	2.5 (1–6)	2.1 (1–3)	287	287	1
Population IIb								
1	1997	65	79	2.9 (1–5)	2.4 (1–3)	191	228	2
2	2000	98	122	2.0 (1–5)	1.6 (1–3)	200	200	2
3	2003	168	155	2.0 (1–5)	2.2 (1–3)	341	341	2

The total number of fish during the fingerling period ranged from 16 169 to 50 962 within each year class. During grow-out in each year class, fish were either kept in the freshwater nucleus station (range: 4459–13 643 fish/year class) or sent to one or two sea test stations (range: 1456–5165 fish/year class, Table 1).

The parents for each generation were selected based on their estimated breeding values (EBV) for growth (since 1992), maturity age (2001), external appearance (2001), skeletal deformations (2002), fillet colour (2003) and cataract caused by *Diplostomum* parasite (2003) (Kause et al. 2005). Parental fish were mated at the Tervo freshwater nucleus station during April–June.

### Rearing conditions

After mating, full-sib egg batches were incubated separately within subdivided trays. At the eyed-egg stage, each full-sib family was transferred to one or two indoor 150-L tanks (Table 1). Eggs hatched in July, and first feeding occurred in August. After two to 3 weeks of growth in the tanks (body weight of 2 g), full-sib families were equalized to similar family size of 150 individuals. Thereafter, the full-sib families were kept separately in 150-L indoor tanks until the start of individual tagging in November. The fingerling period consisted of the first growing season from family size equalization to individual tagging. Individual mortality during the fingerling period was recorded during routine maintenance of fish.

At the size of 50–100 g, fish were individually tagged with Passive Integrated Transponders (Trovan Ltd., Ulm, Germany). After tagging, the fish were either transferred to an outdoor raceway at the freshwater station or sent in April to one or two Baltic Sea test stations (Table 1). One month before transportation, all fish sent to the sea test stations were vaccinated with intraperitoneal injection (1995–1997: 0.1 mL of Lipogen Duo, Aquahealth Ltd, Charlottetown, Canada; 1998–2004: 0.2 mL of Apoject 1800, Pharmaq, Oslo, Norway) against bacterial diseases caused by *Aeromonas salmonica* ssp. *salmonica* and *Listonella (Vibrio) anguillarum*.

Grow-out period began from individual tagging and lasted to the end of the second growing season at sea test stations and to the end of the third growing season at the freshwater station. At the freshwater station, the fish were held in a flow-through earth-bottomed raceway. All sea stations were located in south-west Finland within a maximum distance of 163 km from each other, but locations varied from generation to generation. At the sea stations, the fish were reared under commercial farming conditions in a single net-pen. All fish were fed commercial fish feed pellets throughout the rearing cycle. Survival of the fish was determined after one grow-out season in May at fresh water and in late summer–autumn (July–December) at the sea stations (mean fish weight = 1050 g). In each year class and environment, recording of all fish lasted 2–4 weeks.

### Traits recorded

In total, 14 survival, growth, health, body composition and maturity traits were recorded across life stages and environments. Trait definitions are described in Table 2.

**Table 2:** Measurement unit, measured values, sample sizes (*N*), means, their standard deviations (SD) for traits analysed and year classes during which a trait was recorded

Trait	Unit	Values	N	Mean	SD	Year classes
Survival						
Survival_1_	Proportion	0 = died, 1 = survived	219 951	0.93	0.25	1996–1999, 2001–2002, 2004
Survival_2_	Proportion	0 = died/missing, 1 = survived	81 499	0.72	0.45	1995–2004
Survival_2sea_	Proportion	0 = died/missing, 1 = survived	40 406	0.71	0.45	1999–2004
Body weight						
Weight_1_	g	Continuous	189 299	53.3	21.1	1995–2004
Weight_2_	g	Continuous	58 724	964	310	1995–2004
Weight_3_	g	Continuous	45 242	2374	685	1995–2004
Weight_2sea_	g	Continuous	41 678	1095	335	1996–2004
Health						
Deformation_2_	Proportion	0 = normal, 1 = deformed*	58 781	0.05	0.22	1995–2004
Cataract_2_	Proportion	0 = healthy eyes, 1 = one eye opaque, 2 = both eyes opaque	20 111	0.31	0.63	2001–2004
Body composition						
Flesh colour_2sea_	Score	Categorical (0 = white, …, 30 = dark red)†	5228	29.3	1.9	2001, 2003–2004
Entrail%_2sea_	Percentage	Continuous‡	39 041	11.6	2.0	1996–2001, 2003–2004
Maturity						
Female maturity_2_	Proportion	0 = immature, 1 = mature	20 263	0.61	0.49	1997–2004
Male maturity_2_	Proportion	0 = immature, 1 = mature	21 992	0.25	0.44	1995–2004
Male maturity_2sea_	Proportion	0 = immature, 1 = mature	17 938	0.21	0.41	1996–2004

* Externally visible deformities in head, neck, back or tail.

† Colour of fillet at Róche Salmon colour fan scale (Skrede et al. 1990).

‡ 100 × (Intact body weight − gutted body weight)/Intact body weight.

#### Traits recorded in freshwater station

Fingerling period: Fingerling survival (Survival_1_) was defined as survival between the equalization of families and the start of individual tagging. Individual fish that survived this period were scored as survived (=1), while fish that died were coded as dead (=0). The length of the period from the equalization to the start of tagging varied between year classes (range: 61–147 days). To standardize data collection across families, the end point for Survival_1_ was defined as the time when the first family was tagged. All the fingerlings were individually weighed to the nearest 0.1 g during tagging (Weight_1_) when they had grown for one growing season in fresh water.

Grow-out period: After the second growing season, fish were weighed to the nearest 1 g during April–June (Weight_2_) and their grow-out survival (Survival_2_) between tagging and the end of the grow-out period was recorded. Individual fish that survived between tagging and the end of grow-out period were scored as survived (=1), while fish not present at the end of grow-out period were coded as missing (=0). Fish were classified according to the presence or absence of visually deformed skeletal structures (Deformation_2_). Causative agents of deformations have not been examined, but deformations may be caused by, for example, high water temperature, diseases or deficient composition of a diet (Kause et al. 2007). Fish eye lenses were scored for cataracts caused by parasitic *Diplostomum* spp. eye fluke (Cataract_2_). Furthermore, to generate sex-specific maturity traits, fish were classified after both second and third growing seasons according to their sex and maturity (Male maturity_2_ and Female maturity_2_). Fish whose sex could not be determined or died/missing were coded as missing observations. After the third growing season, fish of unknown sex were late-maturing individuals whose gonads were not visible at this stage, and were coded as immature females. The 3-year-old fish were weighed in late September–November (Weight_3_).

#### Traits recorded in sea stations

After one freshwater growing season (fingerling period) and one sea growing season, fish were weighed to the nearest 1 g during October–April (Weight_2sea_) and their sea grow-out survival between tagging and the end of the grow-out period in sea (Survival_2sea_) was recorded. Male fish were classified to mature and immature (Male maturity_2sea_). No maturity trait could be assigned for females in sea water because fish were harvested before female maturation. At the same time, fillet redness was recorded (Flesh colour_2sea_) using Roché Salmon colour fan scale (Skrede et al. 1990), and gutted body weight was recorded to the nearest 1 g to calculate the percentage of entrails (Entrail%_2sea_ = 100 × (Intact body weight − Gutted body weight)/Intact body weight).

### Genetic analyses

Phenotypic and genetic parameters were estimated using restricted maximum likelihood and multitrait animal models (dmu-ai software; Madsen and Jensen 2008). In the animal model, observations from individual animals and full pedigree are combined, and the degree to which trait(s) covary between all different pairs of an animal’s relatives is used to quantify the amount of genetic (co)variance. Because of the pedigree, genetic correlations can be estimated from individual-level data even between traits that are not recorded from the same individuals, for example in our data a genetic correlation between freshwater and sea water body weight or between survival and harvest body weight (Henderson 1975). The random and fixed factors and the covariates used in the statistical models are presented in Table 3. Full pedigree and all relationships between animals were accounted for in the analysis.

**Table 3:** Statistical models for multitrait animal models

	Random effects		Fixed effects				Covariates
			
Trait	Anim	Year × tank	Year	Year × stat	Year × sex × mat	Year × stat × sex × mat	Tsum (year)
Survival_1_	x	x	x				
Survival_2_	x	x	x				
Survival_2sea_	x	x		x			
Weight_1_	x	x	x				x
Weight_2_	x	x			x		
Weight_3_	x	x			x		
Weight_2sea_	x	x				x	
Deformation_2_	x	x	x				
Cataract_2_	x	x	x				
Flesh colour_2sea_	x	x				x	
Entrail%_2sea_	x	x				x	
Female maturity_2_	x	x	x				
Male maturity_2_	x	x	x				
Male maturity_2sea_	x	x		x			

Model terms are: Anim = genetic effect of an individual with full pedigree; Year × tank = random interaction of birth year and family rearing tank; Year = fixed effect of birth year; Year × stat = fixed interaction of birth year and testing stations in fresh and sea water; Year × sex × mat = fixed interaction of birth year, sex and maturity; Year × stat × sex × mat = fixed interaction of birth year, station, sex and maturity; and Tsum (year) = covariate of cumulative temperature sum at date of recording, nested within birth year.

Asymptotic standard errors for the genetic parameters were computed based on Taylor series approximation (Madsen and Jensen 2008). Genetic correlations of binary traits estimated using linear models are unbiased, whereas phenotypic correlations are biased downwards (Mäntysaari et al. 1991). Residual covariance was always set to zero when calculating genetic correlations between traits that had no records from the same individuals.

The genetic parameters were obtained from one nine-trait run for traits: Survival_1_, Survival_2_, Survival_2sea_, Weight_1_, Weight_2_, Weight_3_, Weight_2sea_, Deformation_2_ and Cataract_2_. For the rest of the traits, the genetic parameters were obtained from separate ten trait runs conducted by adding, one by one, a trait with nine above-mentioned traits. The additive genetic (co)variance matrices were bent to be positive definite using the method of Hayes and Hill (1981).

### Path analysis

Following the studies of Wright (1920), Kause et al. (1999) and Roff and Fairbairn (2011), we used path analysis to visualize the estimated genetic correlations and to generate hypotheses that would explain the observed patterns between survival and growth. The significant genetic correlations between freshwater traits were used as the input in the path analysis, and the mean number of sires and dams in each year class (*n* = 238) were used as the sample size.

In the path analysis, the significant correlations were either maintained as correlations or determined as direct regression paths implying suggested causal relationships. The model construction was initiated by defining a full model in which the correlative or suggested causal relations between all freshwater traits were defined. The sea traits were not included as they were recorded as sib information and did not cover the third growing season. In the full model, freshwater weight and survival traits are genetically correlated because of the direct effects of health traits, cataract and deformations on weight and survival. The path coefficients in the full model were as follows: Weight_1_ directly impacts Deformation_2_, that is, an increase in fingerling weight increases liability to deformations; Deformation_2_ affects Survival_1_, Weight_2_ and Survival_2_; and Cataract_2_ affects Weight_2_, Survival_2_ and Weight_3_. Furthermore, the full model included the following correlations: Weight_1_ was correlated with Weight_2_ and Survival_1_; Survival_2_ was correlated with Survival_1_, Weight_2_ and Weight_3_; Weight_2_ was correlated with Weight_3_; and Deformation_2_ was correlated with Cataract_2_.

After running the full model, we proceeded stepwise by excluding each time the smallest nonsignificant path or correlation and running the reduced model. This was continued until all paths or correlations remained significantly different from zero. The path analysis was carried out with the CALIS procedure and its RAM statement in sas 9.2 (SAS 2008).

## Results

### Genetic correlations: overall trends

**Figure 1:**
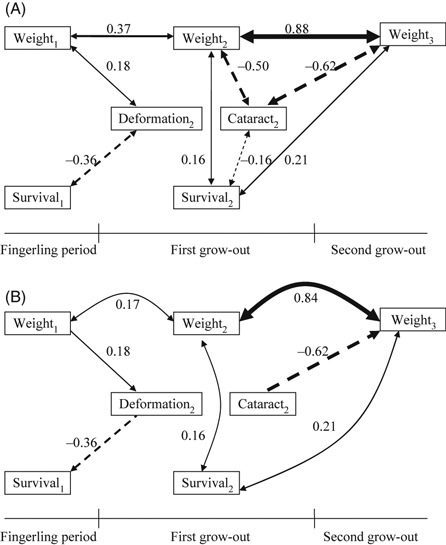
(A) Significant genetic correlations shared by freshwater weight and survival traits. Solid lines denote positive and dashed negative genetic correlations. Width of each line is proportional to the strength of the genetic correlation. (B) The final path diagram for the significant genetic correlations shared by freshwater weight and survival traits. Solid lines denote positive and dashed negative paths. Straight arrows denote direct paths and curved double-headed arrows correlations. Width of each line is proportional to the strength of the path or correlation.

The genetic analyses revealed three broad patterns. First, survival and growth traits were mainly favourably associated genetically, showing that rapid growth enhances survival. This was especially clear during the grow-out period (mean *r*_*G*_ between survival and growth traits = 0.17, range = −0.06 to 0.44). Second, both survival and growth during grow-out were similarly genetically correlated with other traits; for example, increase in the incidence of cataract results in a simultaneous decrease in survival and growth (Fig. 1). Third, the association between growth and fingerling survival was less evident. Fingerling survival was not genetically related to body weight at any age (mean *r*_G_ = −0.07) or to grow-out survival (*r*_G_ range = 0.11–0.14). The genetic and phenotypic correlations between all traits are shown in Appendix 2.

### Growth and survival

Of 12 genetic correlations between body weights and survival traits, four were significantly positive, five positive but nonsignificant and three negative but nonsignificant (Table 4). All significant positive genetic correlations were found between grow-out weights (Weight_2,_ Weight_3_ and Weight_2sea_) and survival (Survival_2_ and Survival_2sea,_Fig. 1A). The correlations between freshwater weights and sea survival were lower than those between freshwater weights and freshwater survival. This is most likely because the causes of mortality differ between environments, making survival an environment-specific trait, and also because growth displays a genotype × environment–interaction (Appendix 2). The fingerling survival (Survival_1_), however, displayed only negative although nonsignificant genetic correlations with body weights (Table 4).

**Table 4:** Genetic correlations ± their standard errors between survival and other traits

Trait	Survival_1_	Survival_2_	Survival_2sea_
Survival_2_	0.13 ± 0.12		
Survival_2sea_	0.10 ± 0.16	**0.56** ± 0.09	
Weight_1_	0.01 ± 0.10	0.10 ± 0.07	0.11 ± 0.10
Weight_2_	−0.14 ± 0.10	**0.16** ± 0.04	−0.05 ± 0.09
Weight_3_	−0.14 ± 0.10	**0.21** ± 0.04	0.07 ± 0.09
Weight_2sea_	0.03 ± 0.11	**0.23** ± 0.07	**0.40** ± 0.08
Deformation_2_	−**0.36** ± 0.14	−0.10 ± 0.09	−0.09 ± 0.14
Cataract_2_	0.07 ± 0.12	−**0.16** ± 0.07	−0.20 ± 0.12
Flesh colour_2sea_	0.25 ± 0.18	0.03 ± 0.13	0.09 ± 0.15
Entrail%_2sea_	0.11 ± 0.10	−0.01 ± 0.06	−0.08 ± 0.07
Female maturity_2_	0.07 ± 0.11	0.06 ± 0.07	−0.05 ± 0.10
Male maturity_2_	−0.07 ± 0.06	0.08 ± 0.08	0.01 ± 0.09
Male maturity_2sea_	−0.12 ± 0.12	0.04 ± 0.09	**0.24** ± 0.11

Correlations significantly different from zero are in bold (95% confidence intervals do not include zero).

### Health traits

Of the six genetic correlations of survival traits with skeletal deformities (Deformation_2_) and parasite-induced cataract (Cataract_2_), two were significantly negative, three negative but nonsignificant and one weakly positive but nonsignificant (Table 4). While all genetic correlations between health and survival traits were in the same direction, the strengths of the genetic correlations differed considerably. Moreover, deformations and cataract were either nonsignificantly (*r*_G_: Deformation_2_ vs. Weight_2_ = 0.05, Deformation_2_ vs. Weight_3_ = −0.07) or moderately strongly negatively, both phenotypically and genetically, correlated with grow-out body weights in fresh water (*r*_G_: Cataract_2_ vs. Weight_2_ = −0.50, Cataract_2_ vs. Weight_3_ = −0.62; *r*_P_: Deformation_2_ vs. Weight_2_ = −0.10, Cataract_2_ vs. Weight_2_ = −0.37, Cataract_2_ vs. Weight_3_ = −0.47). However, in contrast to freshwater grow-out body weights, deformations were positively and significantly genetically correlated with fingerling weight (*r*_G_ = 0.18, Fig. 1A) and positively but nonsignificantly correlated with sea grow-out weights (*r*_G_ = 0.15). Yet, negative genetic correlations between cataract and both fingerling (*r*_G_ = −0.09) and sea grow-out (*r*_G_ = −0.19) weights were found (Appendix 2).

### Body composition traits

The flesh colour was positively and marginally significantly correlated with fingerling survival (*r*_G_ = 0.28). However, there was no genetic relation between flesh colour and grow-out survival in either environment (*r*_G_ = 0.03–0.10). The entrail percentage and survival did not share genetic architecture (*r*_G_ = −0.01 to 0.12). Genetic correlations between entrail percentage and health traits cataract (*r*_G_ = −0.08) and deformation (*r*_G_ = −0.16) were negative but nonsignificant.

### Maturity traits

Male maturity and survival during grow-out in sea environment were significantly genetically correlated (*r*_G_ = 0.27). Thus, the earlier the males matured, the better their survival. Again, this was most likely due to maturity traits being negatively genetically correlated with deformations and cataract (*r*_G_ = −0.30 to −0.15) and strong positive correlations between female maturity and grow-out weights (*r*_G_ = 0.20–0.51). Yet, neither female nor male maturity was genetically significantly correlated with survival in fresh water (*r*_G_ = 0.17–0.24).

### Path analysis

The final path analysis model (Fig. 1B) supported the results from the genetic correlations (Fig. 1A). The main trait connecting growth and survival during the grow-out period was cataract (Fig. 1B). Interestingly, in the path model, cataract had a significant direct path (path coefficient ρ ± standard error = −0.62 ± 0.05) to Weight_3_, but the effect of cataract was not significant towards Survival_2_ (ρ = −0.03 ± 0.05) or Weight_2_ (ρ = 0.20 ± 0.11). However, Survival_2_ remained correlated with both Weight_2_ (*r* = 0.16 ± 0.06) and Weight_3_ (*r* = 0.21 ± 0.07).

Similar to genetic correlations, the paths and correlations of Survival_1_ with all traits other than deformations remained nonsignificant (Fig. 1B). Thus, it is clear that the negative effect (i.e. trade-off) of fast fingerling growth to deformations (ρ = −0.18 ± 0.056) causes fingerling survival not to share genetic architecture with growth or grow-out survival. In addition, the correlation between Weight_1_ and Weight_2_ was lower in final path model (*r* = 0.17 ± 0.03) than the genetic correlation in the initial model (*r*_G_ = 0.37 ± 0.04, Appendix 2), further underscoring the difference between the life stages.

### Heritabilities

Heritabilities for the traits studied have been previously reported (Kause et al. 2002, 2003, 2005; Vehviläinen et al. 2008, 2010a; Kuukka-Anttila et al. 2010). The values obtained in the current study did not substantially deviate from those published earlier (Appendix 1). Heritabilities were low for survival traits (0.07–0.21) and moderate for growth traits (0.27–0.29). Heritabilities were low for Deformation_2_ (0.11) and Flesh colour_2sea_ (0.14), while other traits exhibited moderate-to-high heritabilities (0.27–0.48).

## Discussion

Our results show that, during rainbow trout grow-out, rapid growth is genetically associated with enhanced survival. Thus, our evolutionary null hypothesis that rapid growth imposes a cost on survival is not valid and must be replaced by the alternative hypothesis of overall vigour. The correlation of cataract incidence with both growth and survival provides one explanation for the positive genetic correlation between growth and survival: High cataract incidence is related to hampered growth that reduces grow-out body weights, leading also to lower survival. However, fingerling period survival was not associated with either grow-out survival or any of the growth traits. This was attributable to the genetic cost of increased incidence of skeletal deformations owing to rapid fingerling growth. These contrasting relationships highlight the value of measuring several different traits over several life stages for genetic analyses to avoid missing important associations.

### Growth and survival during grow-out period

In this study, all significant genetic correlations between body weight and survival traits were positive. As a result of fixation of alleles (Roff 1996; Merilä and Sheldon 1999) and resource allocation trade-offs (van Noordwijk and de Jong 1986; McKean et al. 2008), genetic correlations between fitness-related traits are commonly expected to be negative. Although growth is not usually considered as a direct fitness component, rapid growth can be expected to impose a cost on other traits competing for resources especially in stressful conditions (Dmitriew 2011); for example, faster-growing individuals may have lower resistance against specific diseases and parasites. For example, Henryon et al. (2002) found only negative correlations between breeding values for body weights and resistance to highly infectious and fatal viral haemorrhagic septicaemia (*r*_G_ range = −0.33 to −0.14) in rainbow trout. In contrast, our results do not support a trade-off between grow-out growth and overall survival. Instead, the positive genetic correlations found suggest the existence of overall vigour during the grow-out period, that is, fast-growing fish are also the most resistant/tolerant to multiple mortality factors.

Similar to our results, Gitterle et al. (2005) found positive correlations (*r* range = 0.10–0.48) between full-sib family breeding values for harvest body weight and survival in whiteleg shrimp (*Penaeus vannamei*). Positive genetic association between growth and survival has also been found in some terrestrial farm animals. However, these studies on terrestrial livestock have concentrated on very early life stages. For early lamb survival, the genetic correlations with body weight are positive, although quite variable (*r*_*G*_ range = 0.04–0.45; Riggio et al. 2008). In broilers, there is a favourable genetic correlation between body weight and overall mortality (*r*_*G*_ range = −0.15–0.46; de Greef et al. 2001). Birth weight and survival of piglets have shown to be favourably correlated (*r*_*G*_ range = 0.16–0.18; Roehe et al. 2010). Furthermore, the genetic correlation between weaning weight and survival was found to be even stronger (*r*_*G*_ = 0.59; Hellbrügge et al. 2008). There are no estimates for genetic relationship between growth and survival for wild populations. This is because tracking wild individuals over their lifetime to record survival and acquiring their pedigrees are very laborious tasks even with modern tracking and molecular techniques.

In aquaculture generally, and in open farming systems in particular, fish are exposed to diseases and parasites present in the wild, although the capable management minimizes the transmission probabilities. However, owing to stocking density, once a disease or parasite enters an aquaculture population, its effects may be much more drastic than in wild populations.

Parasite-induced cataract is the likely explanation for the positive correlations between growth and survival traits during the grow-out in the population studied. The increased cataract incidence is genetically related to reduced growth, that is, fast-growing fish have less severe cataract. A similar effect was observed by Kuukka-Anttila et al. (2010). The present results revealed further that fish which are genetically resistant and/or tolerant to cataract and grow faster are also more likely to survive. An additional explanation for this overall vigour is that in salmonids, growth rate, feed efficiency and feed intake (proportional to their body weight) are genetically positively associated (Kause et al. 2006; Quinton et al. 2007). This may increase the overall vigour of rapidly growing fish as they have more resources to allocate over competing energy demands. Based on our results, cataract seems to be a key trait connecting growth and survival, yet this conclusion may be specific to our data. A population of fish reared in a parasite-free environment might exhibit different genetic association between growth and survival. Thus, a thorough knowledge of system via extensive trait recording is vital when assessing genetic correlation pattern among growth, survival and underlying third-party traits. This also explains the wide range of survival genetic variance and correlation estimates over space and time (Vehviläinen et al. 2008, 2010a). It is also possible that there exists another unmeasured trait that is associated with growth, survival and cataract and thus caused the observed correlation pattern. Behavioural traits, such as swimming depth or flock social interactions, are possible traits yet to be quantified (Kuukka-Anttila et al. 2010). Nevertheless, the correlation pattern between growth, survival and health traits found in this study does imply that cataract score provides currently the best explanation for overall vigour of fast-growing rainbow trout in Finnish aquaculture conditions.

### Growth and survival during fingerling period

In our study, fingerling body weight and survival did not share genetic architecture either with each other or with grow-out survival. This is because, in contrast to growth during the grow-out period, faster fingerling growth was genetically associated with increased incidence of skeletal deformations. The increase in deformations was further genetically associated with decreased fingerling survival. Although deformations are measured later in life than fingerling survival, it is likely that they are present already in fingerlings. Therefore, the causal paths from rapid fingerling weight to the increase in deformations and from deformations to fingerling survival are logical. Thus, rapid fingerling growth cannot be correlated with high survival.

Faster fingerling growth leading to an increase in skeletal deformations was the only genetic trade-off or cost of rapid growth in the genetic correlation table of 14 traits. The same trade-off between fingerling growth and increase in deformations has been found by Kause et al. (2005) using partly the same data. The genetic cost of rapid fingerling growth did not, however, carryover to reduce grow-out survival. One possibility for this is that the impact of high deformation incidence on reduced survival was restrained by the lower incidence of cataracts in fish that grew well during the grow-out period. Cataracts were strongly related to survival, thus potentially masking the effect of deformations on survival. It can be hypothesized that in a cataract-free population, we might find a trade-off between fingerling growth and survival until harvest. By focusing only on grow-out traits or treating traits recorded in different life stages as one, we would have missed the trade-off between fingerling growth and deformations. This highlights the importance of covering several life stages or periods in genetic studies.

Similar to our results, common carp (*Cyprinus carpio*) displayed a strong positive genetic correlation (0.65) between harvest weight (mean = 1181 g) and survival, but close to zero correlation (0.06) between survival until harvest and weight after one growing season (mean = 144 g) (Nielsen et al. 2010). In an experimental set-up, Lang et al. (2010) showed that juvenile survival after heat shock does not predict survival or weight at harvest in Pacific oyster (*Crassostrea gigas*). The difference in association of growth and survival during different life stages has intrigued a few earlier investigators, although it has not always been possible to extend the analyses into adult stages. Kenway et al. (2006) did not find any consistent pattern (*r*_G_ range = −0.33 to 0.59) between survival and body weight during different periods (0–54 weeks of age) in black tiger shrimp (*Penaeus monodon*). In Atlantic salmon (*Salmo salar* L.), Jonasson (1993) found a positive correlation (*r*_*G*_ = 0.31) between weight and survival within the fingerling period. Rye et al. (1990), on the other hand, found that correlations between fingerling weight and survival exhibited substantial variation among observation periods in Atlantic salmon (−0.11 to 0.45) and rainbow trout (−0.19 to 0.37). In brook trout (*Salvelinus fontinalis*), Robison and Luempert (1984) found that genetic correlations of juvenile survival with egg, fingerling and juvenile weight can even change sign (*r*_*G*_ = 0.37, 0.30 and −0.87, respectively).

### Survival and maturity traits

Early male maturity in the sea environment was genetically associated with better sea grow-out survival, which is not necessarily expected as maturation is an energy-demanding task that draws resources from other body functions (Kiessling et al. 1995). Furthermore, male maturity was not associated with rapid growth at sea. However, the high survival of early-maturing males in the sea environment was, again, explained by the genetic association of early maturity with lower cataract and deformation incidences.

On the other hand, freshwater maturation and survival did not share common genetic architecture. The difference between environments is most likely due to grow-out systems. In earth-bottomed freshwater raceways, a less vigour fish that misses an opportunity to feed, for example owing to more dominant early-maturing fish, can survive by feeding from bottom, which is not possible in free-floating sea net-pens.

The challenge here is that early maturation is not favoured in aquaculture owing to its effects on the energy allocation from muscle growth to gonads, leading to lower quality of fillets. Moreover, maturing fish are aggressive against other fish causing wounding and unequal opportunities for feeding. However, our results do warrant caution in selecting against early maturation as it may increase the incidence of cataract and deformations and further decrease survival at least in the sea environment. In the Finnish rainbow trout breeding programme, the solution has been to select against cataract and deformations especially when selecting for late maturity (Vehviläinen et al. 2010b).

### Survival and body composition

The nonsignificant genetic correlation between survival and percentage of entrails was also unexpected. Percentage of entrails is strongly correlated with the amount of visceral lipid stores (Kause et al. 2007), which are used as an energy source and could be related to enhanced survival via resource allocation. Rainbow trout in the wild utilize lipid stores to survive a long period of starvation during the spawning season (Kiessling et al. 1995). Thus, it can be hypothesized that fish that deposit energy in the form of lipids would have a survival advantage later in the life cycle. However, in an aquaculture setting, the feed is provided in ample amounts. Therefore, fish that utilize the energy for example, during an active defence mechanism, rather than reserve it for later, may have enhanced survival.

Fillet colour on one hand reflects amount of carotenoids, which function as antioxidants and immunostimulants (Wedekind et al. 2008; Lakeh et al. 2010). However, on the other hand, the carotenoids deposited in the flesh may be separate from immunological functions (Blount 2004; Kuukka-Anttila et al. 2010). In this study, we did not find a genetic association between fillet redness and survival. The major correlative traits for enhanced survival were deformations and cataract. It is likely that antioxidants do not play major role in determining the incidence of deformations. Amount of carotenoids could, however, be related to resistance/tolerance to cataracts and correspondingly to survival (Kuukka-Anttila et al. 2010). Indeed, we did find, in contrast to Kuukka-Anttila et al. (2010), that fillet redness was genetically associated with lower incidence of cataracts. However, this association was not strong enough to cascade to a significant direct association between flesh colour and survival traits, although all the genetic correlations between these traits were positive.

### Evolution of genetic correlations

The existence of positive genetic correlations, when negative are expected, has been explained by several phenomena: (i) differences in resource allocation between individuals (van Noordwijk and de Jong 1986), (ii) variation in level of inbreeding between individuals or families (Rose 1984; Phillips et al. 2001), (iii) specific environmental conditions may allow only positive genetic correlations, and genotype-by-environment interactions may switch the sign of genetic correlations (Stearns 1992; Kause et al. 1999, 2001; Kause and Morin 2001; de Jong and Bijma 2002; Lazzaro and Little 2009), (iv) two traits studied may have trade-offs with a third trait (Charlesworth 1990), (v) initially, negative genetic correlation may have disappeared owing to strong selection in the past (Eroukhmanoff 2009) and (vi) a sign of a correlation may depend on the ontogeny/development phase (Lande 1979).

Our study demonstrates that genetic correlation patterns can be explained by a simple framework of including a set of key explanatory traits at different life stages in a quantitative genetic analysis. The results may, however, be specific for our population and the current environment conditions, and care should be taken when extrapolating the results, for example, to newly established trout breeding programmes or programmes for other species. It is possible that long-term selection for growth pushes animals towards a physiological limit, revealing genetic correlations between growth and health traits that would not occur in an unselected population. For instance, if all animals are growing slowly, deformations may be found at low frequency and no correlation would be found between rapid growth and increased deformation incidence. Similarly, long-term selection can deplete genetic variation (Fisher 1930; Merilä and Sheldon 1999). Moreover, mortality factors vary in time and space and across species, and thus, also genetic variation and correlations of survival with other traits will vary greatly (Vehviläinen et al. 2008).

### Practical implications

Genetic trade-offs between growth and disease resistance/tolerance and heath traits such as deformations do exist (this study; Kause et al. 2005; Henryon et al. 2002). Although substantial genetic improvement in growth rate of salmonids has been achieved (Gjøen and Bentsen 1997; Kause et al. 2005; Gjedrem 2010; Rye et al. 2010), the genetic trade-offs are weak enough to allow selection for rapidly growing fish that do not have reduced health. Yet, this requires a routine health recording system and multitrait selection for both production and health traits. For example, Kause et al. (2005) have shown that the amount of deformations can be held at a constant low level while selecting for improved growth rate.

Growth is typically the first trait to be selected in a new selection programme and, in many breeding programmes, the only trait to be selected. Because of the crucial role of survival as an indicator of robustness, adaptation and health, including survival in a selection programme is highly recommended. Recording survival is in fact straightforward in any breeding programme in which initial family sizes are known and individual fish with known pedigree are captured at harvest, allowing breeding value estimation for survival. While recording survival is feasible in many cases, improving survival via selective breeding is challenging. This is because survival is a binary trait, and thus, surviving full-sibs share the same EBV. Accordingly, it would be useful to find continuously distributed traits that are genetically correlated with survival (Vehviläinen et al. 2010b; Kause et al. 2011). Such traits would provide additional information on the genetic potential to survive and generate more accurate EBVs for survival that can differentiate full-sibs.

We showed here that positive genetic correlations exist between survival and production, health and quality traits that can be routinely measured in fish breeding programs. This correlation pattern aids in selection for fish that survive better. Sea survival is economically the most important survival trait in Finnish rainbow trout production. A selection index calculation by Vehviläinen et al. (2010b) showed that direct selection only for sea survival resulted in a selection accuracy of 0.39. Accuracy was elevated to 0.48 when all the traits analysed here together with appearance traits (body shape, skin colour and skin spottiness) are added into the selection index. The most effective appearance trait increasing accuracy of sea survival selection was skin colour. Rainbow trout with more silverish skin are more likely to survive at the sea environment (*r*_G_ = −0.43 ± 0.09, Vehviläinen et al. 2010b). An increase in selection accuracy results in equivalent increase in expected genetic response to selection (Falconer 1960). This means that 22% higher genetic gain in survival is obtained with the help of production and health traits genetically correlated with sea survival.

Survival tends to be a low heritability trait (Fisher 1930; Vehviläinen et al. 2008, 2010a,b), and thus, efforts enhancing its genetic improvement via other traits are valuable. Enhanced survival of farmed fish means less nutrients lost in the sea, better animal welfare and fish adaptation to novel environments, and more affordable protein-rich food produced.

## Data archiving statement

The data used in this study are part of the Finnish national breeding programme for rainbow trout and cannot be made public at this time. Please contact the authors for more information.

## Appendix 1: Variance components of the traits studied

**Table:**

Trait	*V*_G_	*V*_P_	*h*^2^*	*c*^2^
Survival				
Survival_1_	0.003	0.060	0.207	0.103
Survival_2_	0.018	0.196	0.165	0.051
Survival_2sea_	0.007	0.192	0.067	0.043
Body weight				
Weight_1_	78.4	276.0	0.284	0.263
Weight_2_	14 969	54 701	0.274	0.066
Weight_3_	75 731	258 242	0.293	0.059
Weight_2sea_	20 147	70 302	0.287	0.058
Health				
Deformation_2_	0.001	0.045	0.113	0.011
Cataract_2_	0.035	0.221	0.276	0.067
Body composition				
Flesh colour_2sea_	0.206	1.437	0.143	0.032
Entrail%_2sea_	1.373	2.976	0.461	0.014
Maturity				
Female maturity_2_	0.047	0.225	0.335	0.040
Male maturity_2_	0.047	0.179	0.480	0.036
Male maturity_2sea_	0.034	0.147	0.463	0.027

*V*_G_, additive genetic variance; *V*_P_, phenotypic variance; *h*^2^, heritability; *c*^2^, common environment effect.

* Transformed to liability scale for traits: Survival_1_, Survival_2_, Survival_2sea_, Deformation_2_, Cataract_2_, Female maturity_2_, Male maturity_2_, Male maturity_2sea_.

### Appendix 2: Genetic correlations between all other traits

**Table:**

Trait	Weight_1_	Weight_2_	Weight_3_	Weight_2sea_	Deformation_2_	Cataract_2_	Flesh colour_2sea_	Entrail%_2sea_	Female maturity_2_	Male maturity_2_	Male maturity_2sea_
Weight_1_	1	0.41	0.26	0.35	0.01	−0.10	0.04	−0.04	0.11	0.10	0.12
Weight_2_	**0.37** ± 0.04	1	0.71	ne	−0.10	−0.37	ne	ne	0.32	0.05	ne
Weight_3_	**0.24** ± 0.05	**0.88** ± 0.01	1	ne	ne	−0.47	ne	ne	0.60	ne	ne
Weight_2sea_	**0.33** ± 0.05	**0.58** ± 0.04	**0.48** ± 0.05	1	ne	ne	0.41	0.18	ne	ne	0.08
Deformation_2_	**0.18** ± 0.09	0.05 ± 0.07	−0.07 ± 0.07	0.15 ± 0.09	1	0.04	ne	ne	ne	ne	ne
Cataract_2_	−0.09 ± 0.07	−**0.50** ± 0.06	−**0.62** ± 0.05	−**0.19** ± 0.07	0.08 ± 0.11	1	ne	ne	−0.13	0.03	ne
Flesh colour_2sea_	0.00 ± 0.11	0.17 ± 0.11	**0.24** ± 0.11	**0.20** ± 0.10	−0.11 ± 0.17	−**0.29** ± 0.12	1	0.16	ne	ne	0.01
Entrail%_2sea_	−**0.09** ± 0.05	**0.11** ± 0.05	0.05 ± 0.05	**0.17** ± 0.05	−0.16 ± 0.08	−0.08 ± 0.07	**0.31** ± 0.09	1	ne	ne	0.11
Female maturity_2_	−0.07 ± 0.06	**0.33** ± 0.05	**0.51** ± 0.05	**0.20** ± 0.06	−**0.20** ± 0.10	−**0.18** ± 0.08	0.00 ± 0.12	**0.17** ± 0.05	1	ne	ne
Male maturity_2_	0.01 ± 0.06	0.08 ± 0.05	0.08 ± 0.04	0.11 ± 0.06	−**0.27** ± 0.08	−0.15 ± 0.09	−0.10 ± 0.13	0.02 ± 0.05	**0.74** ± 0.04	1	ne
Male maturity_2sea_	0.06 ± 0.07	0.13 ± 0.07	0.12 ± 0.07	0.12 ± 0.07	−**0.30** ± 0.10	−**0.21** ± 0.10	−0.02 ± 0.13	0.10 ± 0.06	**0.71** ± 0.04	**0.92** ± 0.02	1

ne = nonestimable – not recorded from same animals.

Genetic correlations ± their standard errors below and phenotypic correlations above diagonal. Genetic correlations significantly different from zero are in bold.
